# A Biocompatible Aspartic-Decorated Metal–Organic Framework with Tubular Motif Degradable under Physiological Conditions

**DOI:** 10.1021/acs.inorgchem.1c01701

**Published:** 2021-09-02

**Authors:** Marta Mon, Rosaria Bruno, Rosamaria Lappano, Marcello Maggiolini, Leonardo Di Donna, Jesus Ferrando Soria, Donatella Armentano, Emilio Pardo

**Affiliations:** † Departament de Química Inorgànica, Instituto de Ciencia Molecular (ICMOL), Universitat de València, 46980 Paterna, València, Spain; ‡ Dipartimento di Chimica e Tecnologie Chimiche, Università della Calabria, Rende 87036, Cosenza, Italy; § Dipartimento di Farmacia e Scienze della Salute e della Nutrizione, Università della Calabria, Rende 87036, Cosenza, Italy

## Abstract

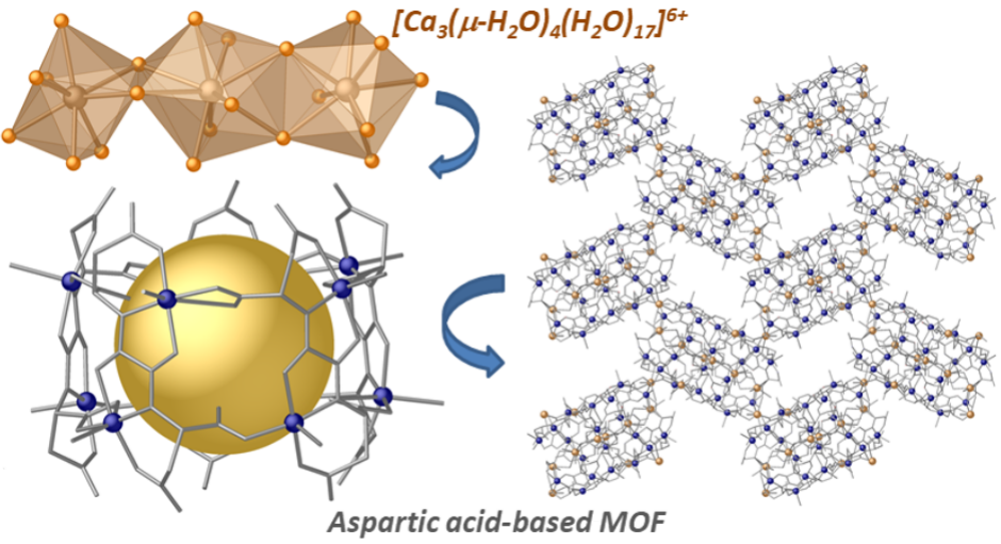

Achieving a precise control of the final structure of metal–organic frameworks (MOFs) is necessary to obtain desired physical properties. Here, we describe how the use of a metalloligand design strategy and a judicious choice of ligands inspired from nature is a versatile approach to succeed in this challenging task. We report a new porous chiral MOF, with the formula Ca_5_^II^{Cu^II^_10_[(*S*,*S*)-aspartamox]_5_}·160H_2_O (**1**), constructed from Cu^2+^ and Ca^2+^ ions and aspartic acid-decorated ligands, where biometal Cu^2+^ ions are bridged by the carboxylate groups of aspartic acid moieties. The structure of MOF **1** reveals an infinite network of basket-like cages, built by 10 crystallographically distinct Cu(II) metal ions and five aspartamox ligands acting as bricks of a tubular motif, composed of seven basket-like cages each. The pillared hepta-packed cages generate pseudo-rhombohedral nanosized channels of ca. 0.7 and 0.4 nm along the *b* and *a* crystallographic axes. This intricate porous 3D network is anionic and chiral, each cage displaying receptor properties toward three-nuclear [Ca_3_(μ-H_2_O)_4_(H_2_O)_17_]^6+^ entities. **1** represents the first example of an extended porous structure based on essential biometals Cu^2+^ and Ca^2+^ ions together with aspartic acid as amino acid. **1** shows good biocompatibility, making it a good candidate to be used as a drug carrier, and hydrolyzes in acid water. The hypothesis has been further supported by an adsorption experiment here reported, as a proof-of-principle study, using dopamine hydrochloride as a model drug to follow the encapsulation process. Results validate the potential ability of **1** to act as a drug carrier. Thus, these make this MOF one of the few examples of biocompatible and degradable porous solid carriers for eventual release of drugs in the stomach stimulated by gastric low pH.

## Abstract 2

We report the rational design of a new biocompatible and eco-friendly chiral *bio*MOF. It represents a rare example of a MOF constructed from ligands and metal ions of low toxicity.

## Introduction

Metal–organic frameworks (MOFs)^1−3^ have become a hot topic of research during the past decades due to the concomitant presence of atheistically pleasant porous high-dimensional structures with intriguing net topologies and thrilling chemical and physical properties. Indeed, these hybrid materials—consisting of metal ions (or small metal clusters) linked by a wide diversity of organic spacers—have been able to make themselves a functional entity by controlling the assembling of their subunits, which have enabled them to find applications in gas storage and separation,^4−6^ drug delivery,^7−9^ molecular recognition,^10−^^,14^ and catalysis^15−18^ as well as in templating the *in situ* growth/encapsulation of a wide variety of functional moieties^19,20^

Biomedical applications of MOFs are still in their infancy, but encouraging results have been developed in the past years. A quite recent step is the bioengineering of MOFs for the design of original examples of MOFs using biomolecules that constitute the *bricks* of life. This subclass of materials, referred to as *bio*MOFs,^21,22^ can be obtained using ligands derived from simple amino acids,^23^ nucleobases^24^ and aminosaccharides,^25^ or their larger derivatives polypeptides,^26^ polynucleotides, and polysaccharides, and even small proteins,^27^ nucleic acids, or complex sugars (like glycogen). *Bio*MOFs may offer remarkable advantages over traditional MOFs: (i) the possibility to achieve homochiral solids crystallizing in polar space groups in a rational and predictable way with applications in chiral discrimination or separation, due to the enantiopure nature of many biomolecules, (ii) large biocompatibility and feasibility in medical applications, (iii) increased stability in hydrated environments, and (iv) molecular recognition capabilities reminiscent of biological processes. However, despite their remarkable features, the strategy based on the direct synthesis of *bio*MOFs with open-framework structures capable to host and then align in their confined spaces the guest molecules/ions required to develop new biomimetic scaffolds has been barely explored, and only a limited number of MOFs of established biocompatibility have been reported^28−30^ Thus, more work is required to further increase our understanding on such bio-mimicking host-guest interactions, and consequently, open in the near future a myriad of potential biotechnological applications.

**Scheme 1:**
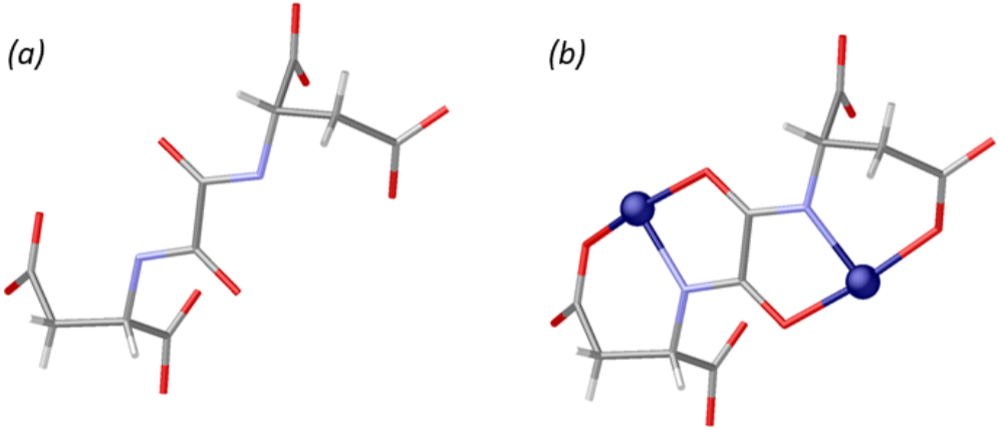
Chemical Structure of the Chiral Bis(dimethylaspartate)oxalamide Ligand H_2_Me_2_-(*S*,*S*)-aspartamox (a) and the Corresponding Dicopper(II) Motif Building the MOF (b)

In this context, aiming to make a step forward on current limitations of *bio*MOFs and prompted by their properties, our research efforts have been devoted to develop a programmed strategy for the rational design of these materials focused on a family of enantiopure disubstituted oxamidato ligands derived from natural amino acids.^31,32^ In particular, we have been able to obtain different examples of three-dimensional *bi*oMOFs, where their functionality has been directed by the chemical nature of the residue of the constituent amino acid. Extending the application of this concept, here we report the synthesis of a novel chiral oxamidato-based *bio*MOF, of formula Ca_5_^II^{Cu^II^_10_[(*S*,*S*)-aspartamox]_5_}·160H_2_O (**1**), (H_2_Me_2_-(*S*,*S*)-aspartamox = bis[(*S*)-dimethylaspartate]oxalyl diamide, Scheme 1) prepared from a ligand derived from the natural amino acid l-aspartic acid, which displays receptor properties toward three-nuclear calcium(II) entities—where Ca^2+^ ions are highly solvated, mimicking the environment of biological systems.^33−35^**1** represents a potential playground to gain insight and, later on, try to mimick the possible binding sites of the complex supramolecular assemblies acting in many biological Ca^2+^ dependent processes, such as in the Ca^2+^ binding proteins, Calmodulin (CaM). In addition, the biocompatibility of **1** has been demonstrated with MTT assays on MCF7 and SkBr3 breast cancer cells, which together with their degradability under physiological conditions and confirmed capability to load small drug’s molecules, open the way for the future application of **1** for drug delivery.

## Synthesis and X-ray Crystal Structure

We report herein the application of the metalloligand design strategy to obtain a novel biocompatible and water-stable 3D MOF of formula Ca_5_^II^{Cu^II^_10_[(*S*,*S*)-aspartamox]_5_}·160H_2_O (**1**), which was obtained as blue irregular prisms by slow diffusion of aqueous solutions containing stoichiometric amounts of (Bu_4_N)_2_{Cu_2_[(*S*,*S*)-aspartamox](OH)_2_}·4H_2_O and CaCl_2_ in H-shaped tubes at room temperature.

**Figure 1:**
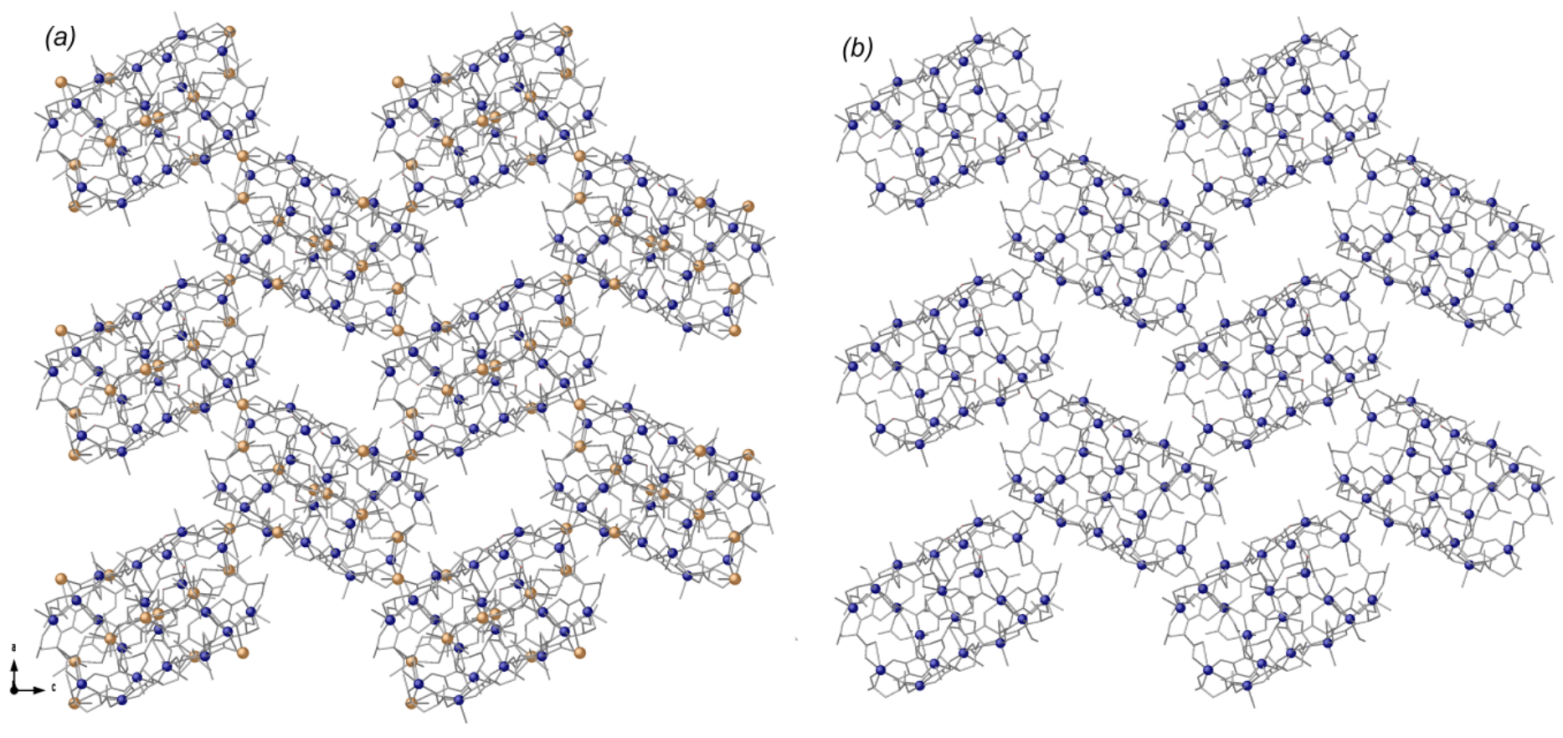
A portion of the crystal structure of **1** viewed along the [010] direction: (a) The intricate porous 3D network generated by the interconnection of “helices of cages” via four aspartic acid residues from adjacent ones. Each cage encapsulates [Ca_3_(μ-H_2_O)_4_(H_2_O)_17_]^6+^. In (b), calcium clusters have not been depicted to show the overall Cu(II) 3D network. Copper and calcium atoms are represented by blue and gold spheres, respectively, whereas the ligands are depicted as sticks.

**Figure 2:**
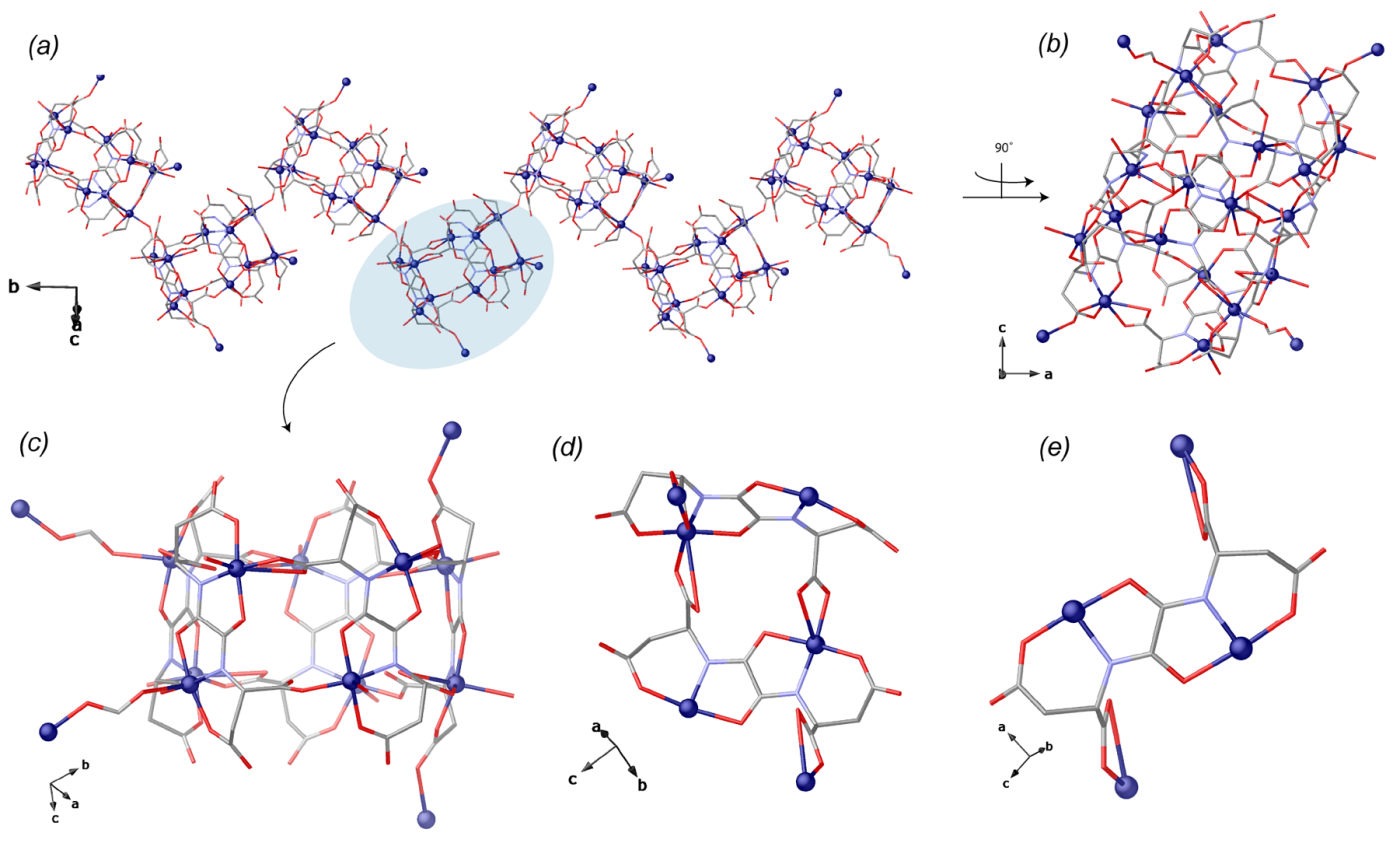
(a) Perspective view of hepta-packed cages generating helices, interconnected through carboxylic functions of the aspartic acid residues with four other aspartic acid moieties by means of bridging carboxyl and hydroxyl groups from adjacent ones; (b) view of one helix along the *b* crystallographic axis; (c) perspective view of basket-like cages built by 10 crystallographically distinct Cu(II) metal ions and 5 aspartamox; (d, e) details on ligand coordination mode within each cage. Copper atoms are represented by blue spheres, whereas the ligands are depicted as blue and red sticks for nitrogen and oxygen bonds, respectively.

**Figure 3:**
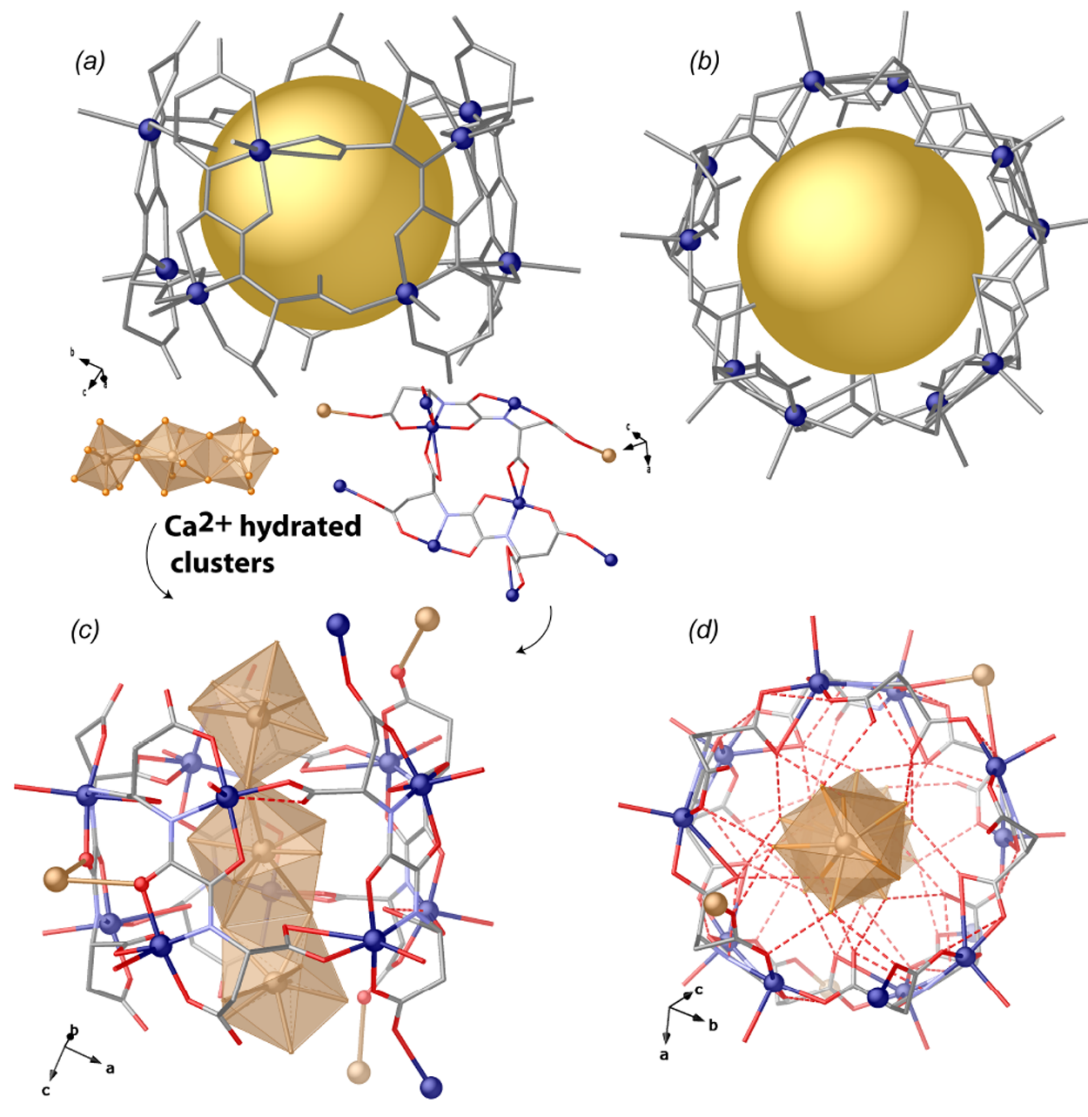
Details on Cu_10_ cages: (a, b) side and top views of cages featuring a narrow window with a van der Waals diameter of ca. 8 Å and an effective cage size of 268 Å^3^ (highlighted by gold spheres); (c, d) side and top views of Cu_10_ cages embedding [Ca_3_(μ-H_2_O)_4_(H_2_O)_17_]^6+^ perfectly recognized by means of H-bonds. Copper and calcium atoms are represented by blue and gold spheres or polyhedra, respectively, whereas the ligands are depicted as blue and red sticks for nitrogen and oxygen bonds, respectively. O···O_W_ hydrogen bonds have been depicted with red dashed lines, respectively.

The crystal structure of **1** determined by single-crystal X-ray diffraction unveiled that it crystallizes in the chiral *P*2_1_2_1_2_1_ space group of the orthorhombic system, with an absolute structure parameter (Flack calculated with Parsons method)^36^ of 0.046(5). The structure of **1** reveals an infinite network of basket-like cages presenting a narrow window with a van der Waals diameter of ca. 8 Å and a cage size of ca. 268 Å^3^ (Figures 1–3). The asymmetric unit is built by 10 crystallographically distinct Cu(II) metal ions and five aspartamox ligands (Figures 2c, 3, and S1), together with five distinct Ca^2+^ metal ions and a huge amount of water molecules (Figure S1). Each Cu_10_ cage is further interconnected through hydroxyl functions of the aspartic acid residues (Figure 2a,c) yielding left-handed helices, as bricks of a tubular motif, composed of seven basket-like cages each (Figure 2a,b) developing along the [010] direction. The intricate anionic, chiral, and porous 3D network is generated by the interconnection of those helices with four aspartic acid residues by means of bridging carboxyl and hydroxyl groups from adjacent ones (Figure 1). Thus, the pillared hepta-packed cages generate two nanosized channels growing along the *a* (Figure S2) and *b* crystallographic axes (Figure 1 and Figure S3). The latter unveil an irregular shape, pseudo-rhombohedral, exhibiting a virtual diameter of ca. 0.7 nm. The other one has a more squared shape with ca. 0.4 nm as virtual diameter. As shown in Figures S2 and S3, both channels are decorated by coordinated water molecules and oxygen atoms from the ligand’s moieties, giving them a hydrophilic character. It might also explain the large number of water molecules embedded within the structure, together with its capability to exchange them loading dopamine hydrochloride drug, likely stabilized by hydrogen bonds supramolecular interactions.

In **1**, each cage hosts three-nuclear [Ca_3_(μ-H_2_O)_4_(H_2_O)_17_]^6+^ entities [Ca–O distances in the range of 2.320(3)–2.874(4) Å] (Figure 3c,d), where Ca^2+^ ions are solvated, being surrounded by eight [Ca(1) and Ca(3)] or nine [Ca(2)] water molecules. Further Ca^2+^ metal ions reside in the interstitial voids and interact with the carboxyl fragment of aspartic acid residues, contributing to interconnect the basket-like Cu_10_ cages (Figures 1b and 3c,d). The position of the calcium clusters in the cages of **1** unveils that it occurs through a molecular recognition process, governed by H-bonds involving water molecules surrounding alkaline-earth metal ions and carboxyl and hydroxyl groups of the aspartic acid moieties (Figure 3d). The 10 crystallographically distinct Cu^2+^ metal ions adopt highly distorted octahedral (CuN_2_O_3_O_water_ or CuNO_4_O_W_ or CuN_3_O_2_O_w_) coordination geometries (Figures 2a and S1). The aspartamox ligand exhibits a symmetric coordination mode, involving COO^–^ groups in metal binding. It coordinates Cu^2+^ and Ca^2+^ metal cations, producing up to five different environments for copper(II) ions (Figures 1 and S4).

The *cis* oxamidato-bridged dicopper(II) units, {Cu^II^_2_[(*S*,*S*)-aspartamox]}, coordinate each other through bridging carboxyl and hydroxyl groups in a zipper-like fashion (Figure 2c,d). Taking a more in-depth look, each as-made dimer, featuring two side-chains of aspartic acid, encompasses one carboxyl for intradimer linking together with the hydroxyl one acting as a linker to adjacent {Cu^II^_2_[(*S*,*S*)-aspartamox]} moieties (Figures 2d,e and S4a) in an *interlocking* fashion (Figure 2d). The additional coordination of those residues with carboxyl “free” groups toward Cu(II) (Figure S4b) or Ca(II) ions (Figure S4c) ensures the cohesion of adjacent interlocked {Cu^II^_4_[(*S*,*S*)-aspartamox]_2_} units (Figure 2b,c), self-assembling 10 copper ions unfolding the chiral basket-like cage. Noteworthy, not all those free carboxyl terminations coordinate metal ions; a portion of them decorate the two gateways of the cages being stabilized by solvent molecules H-bonded to the cages. The two gateways of the cages can be described as *ribbons* of copper metal ions surrounded by aspartic acid residues pointing toward metal ions or water molecules, while the barriers of the cage are delineated by a robust oxamate core of the aspartamox ligand (Figure 3c). Each Cu_10_L_5_ as-made cage is four-linked by four O atoms from aspartic residues acting as *cluster’s connectors* of adjacent *baskets* (Figure 2a). Six-coordinated Cu metal centers have similar Cu–O and Cu–N bond lengths, either for the basal/equatorial planes [1.947(6)–2.049(6) and 1.916(8)–1.971(7) Å] or for apical ones [Cu–O: 2.337(6)–2.980(7) Å], typical of axial elongated distortions as expected.

The basket-like cages show exceptional receptor properties through multiple H-bonding interactions toward [Ca_3_(μ-H_2_O)_4_(H_2_O)_17_]^6+^ hydrated clusters, which occupy the centers of the holes (Figure 3). They are linked to the wall of the toric-like anionic cage by means of strong hydrogen bonds stabilizing the huge hydrated calcium environment reminiscent of binding in Calmodulin. In this ubiquitously expressed Ca^2+^-binding protein, the coordination to Asp amino acid is typically mediated by water molecules that are hydrogen-bonded to the side chain of the amino acid or through the backbone of amino acids in general. In **1**, it occurs thanks to oxygen atoms from the terminal side of the aspartic acid residues and oxamate ligand’s core, which act as *hooks* to anchor guest moieties to the cage’s wall [O_W_···O distances varying in the range 2.74–2.98 Å].

The structural analysis unveiled the occurrence of a large number of crystallization water molecules (not modeled) placed inside the hydrophilic channels developing along the *a* and *b* axes. The resulting porous structure facilitates the adsorption/desorption of the solvent from the crystal, as shown by thermogravimetric analysis, but this process occurs with the loss of the crystallinity. Without found solvent molecules, the effective free volumes of **1** are calculated by PLATON analysis to be 55% of the crystal volume (9548.6 Å^3^ of the 17355.0 Å^3^ of the unit cell volume). In accordance with SCXRD analysis, the channels of **1** are entirely filled by solvent guests (**1**) interacting and stabilizing [Ca_3_(μ-H_2_O)_4_(H_2_O)_17_]^6+^ hydrated clusters (Figure 3d and crystallographic details in the Supporting Information).

**Figure 4:**
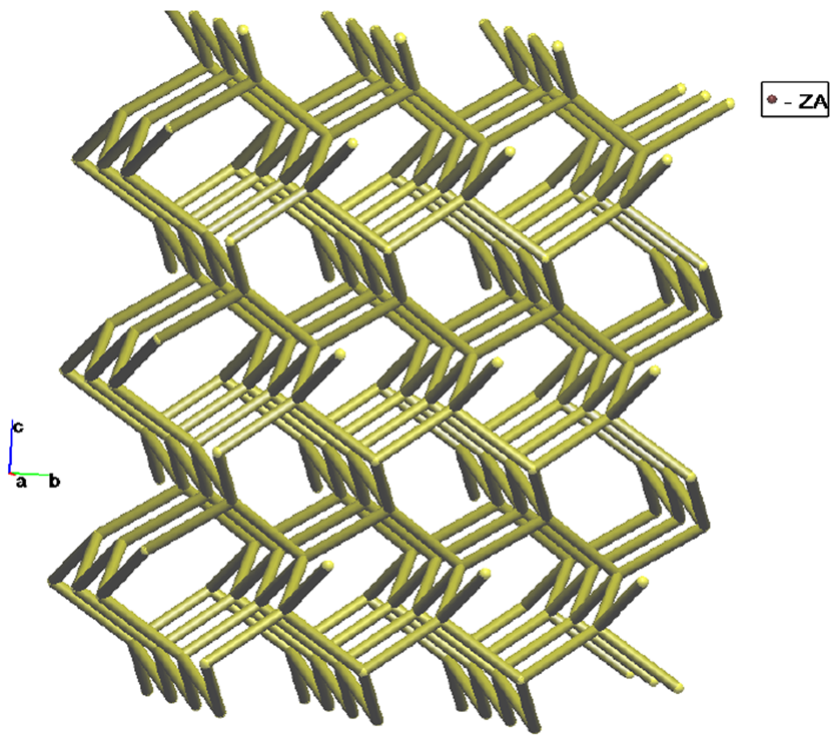
Schematic view of the **dia** net topology in **1**.

The crystal structure of **1** was deconstructed by applying the concept of the simplified underlying net and also using TOPOSPRO software in order to get a brief topological analysis. So, the basket-like cages (Figures S5–S7) can be represented as a node connected to adjacent nodes at a distance of ca. 15 Å. Thus, the overall structure unfolds a **dia** net (Figures 4 and S5–S7).

## Thermogravimetric Analysis and X-ray Powder Diffraction

The water content of **1** was determined by thermogravimetric analysis (TGA) under a dry N_2_ atmosphere. It shows a fast mass loss from room temperature to *ca*. 420 K, followed by a plateau in the mass loss until decomposition starts. The estimated percentage weight loss value of 43% (Figure S8 in the Supporting Information) corresponds to 130 H_2_O molecules. This slightly mismatch between estimated solvent molecules by TGA and SQUEEZE total count of electrons (*vide infra*) is most likely due to the fast loss of solvent, often shown by such porous materials. The experimental powder X-ray diffraction (PXRD) patterns of a polycrystalline sample of **1** confirm the purity and homogeneity of the bulk sample (Figure S9a,b) and that the 3D anionic network does not experience significant phase transitions in the range of 100–298 K.

## Biocompatibility and Degradability Properties

### MTT Assay

**Figure 5:**
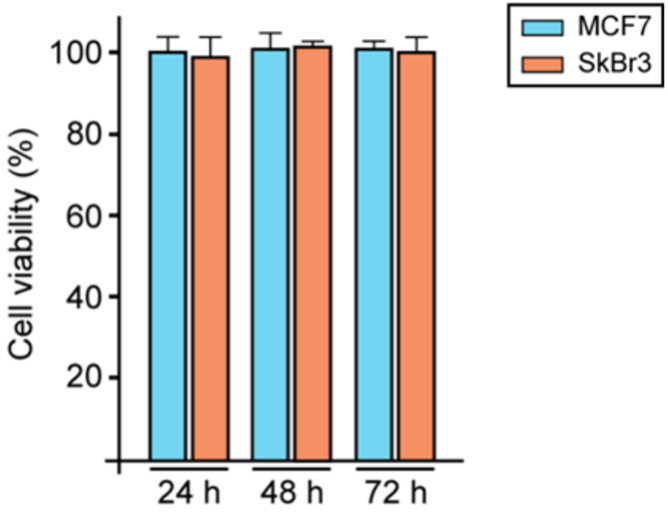
Viability of MCF7 and SkBr3 breast cancer cells upon exposure to 5 μM of *bio*MOF **1** was assessed by MTT assay and expressed as a percentage of cells exposed to chemicals with respect to vehicle treated cells.

The effects of **1** on cell viability were determined by the MTT [3-(4,5-dimethylthiazol-2-yl)-2,5-diphenyltetrazolium bromide] assay. It is based on the conversion of MTT to MTT formazan by mitochondrial enzyme. Cells were seeded in quadruplicate using 96-well plates in regular growth medium and grown until 70% confluence. After that, cells were washed once they had attached and then treated with 5 μM solution of **1** in regular growth medium. Relative cell viability was determined after 24, 48, and 72 h by MTT assay according to the manufacturer’s protocol (Sigma-Aldrich, Milan, Italy). The cell viability was expressed as a percentage of cells exposed to chemicals with respect to vehicle treated cells. Remarkably, **1** for 72 h did not alter cell viability (Figure 5). These findings, even if preliminary, suggest the good biocompatibility of **1** making it as a good candidate as a drug nanocarrier.

### Loading Experiment

**Figure 6:**
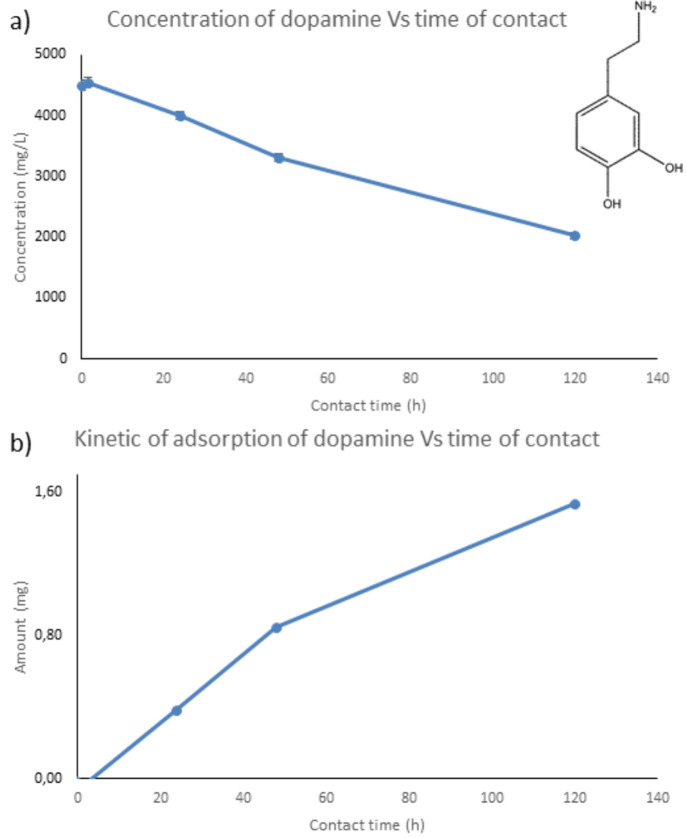
(a) Concentration of dopamine hydrochloride in the soaked solution and (b) the adsorption amount in mg of drug versus time in 8.25 mg of polycrystalline powder of MOF, which has been soaked in a solution of dopamine hydrochloride at 4510 mg/L (*V* = 0.55 mL). Inset in (a) shows the scheme of dopamine drug.

In order to test the ability of the MOF to act as a drug carrier, an adsorption experiment has been set up, as a proof-of-principle study of guest inclusion, using dopamine hydrochloride as model drug for the encapsulation process. 8.25 mg of MOF was soaked in 0.55 mL of a solution of dopamine hydrochloride at 4510 mg/L. The concentration of the solution was monitored by high performance liquid chromatography ultraviolet (HPLC/UV) by picking up 30 μL at different times (Figures 6 and S10). Figure 6a shows the decreasing of concentration of dopamine in the contact solution over a time period of 5 days. The kinetics of the adsorption is shown in Figure 6b, which describes the amount of dopamine adsorbed by the MOF powder. It is worth to note that the amount of dopamine adsorbed by the MOF after 5 days is around 62% of the initial loading, providing an adsorption capacity of the material of 0.186 g of drug per gram of solid at the concentration studied.

### Degradability Properties and pH Dependence Studies

To investigate the degradability of **1** under gastric physiological conditions, the dissolution of 1 g of powder of **1** in 100 mL of acidic water–solution at pH = 2 has been followed. After 2 h, the total degradation of **1** has been observed, going along with chromatic change of the aqueous acidic medium to a light blue solution, after release of Ca^2+^—followed by inductively coupled plasma mass spectrometry (ICP-MS, see Experimental Section and Figure S11)—and Cu^2+^ ions. To further investigate the structural stability in aqueous media, we performed PXRD in acidic (pH = 3), pure water, and basic aqueous media (pH = 11) by immersing powder of **1** in aqueous solutions for a given time (Figure S9c–e). As shown in Figure S9c,d, the PXRD patterns of a polycrystalline sample of **1** show retention of crystallinity over the range of pH studied. This confirmed the good structural robustness of the material in water and its acidic lability. Indeed, at pH = 3 (Figure S9e) and lower, the release of drug can be accomplished through the degradation of the biocompatible framework (see Figure S11).

This represents only an initial result to place **1** among the valuable candidates for drugs encapsulation and their further release in acidic conditions, without any implication of recovery process or toxic effects, thanks to its biocompatibility.

## Conclusions

Here, we have rationally designed a new biocompatible chiral *bio*MOF. It represents a rare example of a MOF constructed from nontoxic ligands and using biometals,^22^ copper, and calcium ions. An aspartic acid-based ligand has been used to build a novel porous network, which shows the capability to encapsulate dopamine and degrades under acidic gastric physiological conditions. The use of coordinating units, built by metal ions and organic linkers, of minimal toxicity should be considered when constructing MOFs as platforms for drug carriers or therapeutic agents. The results here presented of dopamine hydrochloride loading as a drug model represent a promising start for **1** to act as a drug carrier in more realistic/physiological conditions. These results represent a step forward to efficiently exploit MOFs for the transport of chiral drugs, a field of paramount importance, where MOFs have started to show they can be game changers. Further work is currently developed in our lab in this direction.

## Experimental Section

### Materials

All chemicals were of reagent grade quality. They were purchased from commercial sources and used as received.

### Physical Techniques

Elemental (C, H, N) analyses were performed at the Microanalytical Service of the Universitat de València. FT-IR spectra were recorded on a PerkinElmer 882 spectrophotometer as KBr pellets. ^1^H NMR spectra were recorded at room temperature on a Bruker AC 200 (200.1 MHz) spectrometer. Thermogravimetric analysis (TGA) data were recorded on an SDT-Q600 analyzer from TA Instruments. The temperature varied from RT to 600 °C at a heating rate of 10 °C·min^–1^. Measurements were carried out on samples in open platinum crucibles under a flow of air.

### Preparation of H_2_ Me_2_-(*S*,*S*)-aspartamox = Bis[(*S*)-dimethylaspartate]oxalyl Diamide

The proligand was prepared using the following synthetic procedure: First, under a N_2_ atm, an excess of thionyl chloride (13.10 mL, 180 mmol) was added dropwise, under stirring at 0 °C on an ice bath, to a solution of (l)-aspartic acid (7.99 g, 60 mmol) in 150 mL of MeOH. The resulting colorless solution was refluxed for 6 h and kept under stirring overnight. Then, the excess of thionyl chloride was distilled with MeOH (3 × 150 mL). The reaction mixture was washed with acetone (150 mL) and diethyl ether (100 mL) and further concentrated, under reduced pressure, to afford the dimethyl ester derivative of the (l)-aspartic acid amino acid, which was used in the next step without further purification. Second, the resulting dimethyl ester derivative of the (l)-aspartic acid amino acid (9.67 g, 60 mmol) was dissolved in 250 mL of dichloromethane and charged with triethylamine (8.4 mL, 60 mmol). To the resulting colorless reaction mixture was added dropwise another solution containing oxalyl chloride (2.54 mL, 30.0 mmol) in dichloromethane (50 mL) under stirring at 0 °C on an ice bath. The resulting solution was stirred overnight. The small amount of white solid (Et_3_NHCl) obtained was filtered off, and the resulting solution was concentrated to dryness in a rotatory evaporator to afford a white solid, which was suspended in tetrahydrofuran to extract the target proligand and remove the remaining Et_3_NHCl and then further concentrated in a rotatory evaporator and dried under vacuum. Yield: 10.16 g, 90%; Anal. Calcd (%) for C_14_H_20_N_2_O_10_ (376.3): C 44.68, H 5.36, N 7.44; found C 44.36, H 5.26, N 7.36; ^1^H NMR (CDCl_3_): 2.88 (dd, 2H; CH_2_), 3.10 (dd, 2H; CH_2_), 3.73 (s, 3H; OCH_3_), 3.81 (s, 3H; OCH_3_), 4.86 (t, 2H; CH), 8.20 (d, 2H; NH from CONH). IR (KBr): ν = 1760, 1740, and 1650 cm^–1^ (C=O).

### Preparation of (Me_4_N)_2_{Cu_2_[(*S*,*S*)-aspartamox](OH)_2_}·4H_2_O

An aqueous solution (60 mL) of H_2_ Me_2_-(*S*,*S*)-aspartamox (11.29 g, 30 mmol) was treated with a 25% methanolic solution of Me_4_NOH (150 mmol). Another aqueous solution (35 mL) of CuCl_2_·2H_2_O (10.23 g, 60 mmol) was then added dropwise while the reaction mixture was stirred. The resulting deep green solution was filtered to remove solid particles and then concentrated to a volume of ca. 10 mL in a rotary evaporator. The mixture was then allowed to stand at 0 °C on an ice bath for 15 min, and finally, it was filtered to afford a green polycrystalline solid that was gently washed with acetone, filtered off, and dried under vacuum. Yield: 12.31 g, 59%; Anal. Calcd for C_18_H_40_Cu_2_N_4_O_16_ (695.62): C, 31.08; H, 5.80; N, 8.05%. Found: C, 30.97; H, 5.73; N, 8.12%; IR (KBr): ν = 3641 cm^–1^ (O-H), 2986 cm^–1^ (C-H), 1708, 1643, and 1619 cm^–1^ (C=O).

### Preparation of Ca_5_^II^{Cu^II^_10_[(*S*,*S*)-aspartamox]_5_}·160H_2_O (**1**)

Well-shaped elongated prisms of **1** appropriate for X-ray structural analysis were obtained by slow diffusion in an H-shaped tube containing aqueous solutions of stoichiometric amounts of (Me_4_N)_2_{Cu_2_[(*S*,*S*)-aspartamox](OH)_2_}·4H_2_O (0.100 g, 0.1 mmol) in one arm and CaCl_2_ (0.011 g, 0.1 mmol) in the other. Anal. Calcd for C_50_Cu_10_Ca_5_H_350_N_10_O_210_ (5289.11): C, 11.35; H, 6.67; N, 2.65%. Found: C, 11.28; H, 7.05; N, 2.67%; IR (KBr): ν = 1602 cm^–1^ (C=O).

### Response of Ca_5_^II^{Cu^II^_10_[(*S*,*S*)-aspartamox]_5_·160H_2_O (**1**) to Gastric Low pH

1 g of powder of **1** has been treated with 100 mL of acidic water solution at pH = 2. After 2 h has been observed the total degradation of **1** (see Figure S11).

### X-ray Powder Diffraction Measurements

A fresh polycrystalline sample of **1** was introduced into 0.5 mm borosilicate capillaries prior to being mounted and aligned on a Bruker D8 Discover powder diffractometer, using Cu Kα radiation (λ = 1.54056 Å). Five repeated measurements were collected at room temperature (2θ = 2–50°) for each sample and merged in a single diffractogram. The simulated powder pattern was calculated from single-crystal X-ray diffraction data and processed by the Mercury program (Version 4.2.0)^37^ provided by the Cambridge Crystallographic Data Centre.

### Single-Crystal X-ray Diffraction

A crystal of **1** was selected and mounted on a MITIGEN holder in Paratone oil and then quickly placed on a liquid nitrogen stream cooled at 90 K to avoid the degradation upon dehydration. Diffraction data were collected on a Bruker-Nonius X8APEXII CCD area detector diffractometer using graphite-monochromated Mo-Kα radiation (λ = 0.71073 Å). The data were processed through the SAINT reduction and SADABS^38^ multi-scan absorption software, and the structure was solved with the SHELXS structure solution program, using the Patterson method. The model was refined with version 2018/3 of SHELXL against *F*^2^ on all data by full-matrix least-squares.^39,40^

All non-hydrogen atoms were refined anisotropically with the use of restrains on geometrical (DFIX) and displacement parameters (SIMU and DELU). Some additional restrains to make the refinement more efficient have been applied; for instance, ADP components have been restrained to be similar to other related atoms, using SIMU 0.04 for disordered fragments or EADP for a group of atoms of the ligands expected to have essentially similar ADPs. The solvent molecules were disordered (some refined double positions O27W, O28W, and O78W), and they have been only in part modeled. The quite large channels featured by these MOF likely account for that. In fact, only water molecules pseudo-coordinated to copper and calcium metal ions have been modeled. Any attempt to locate and model the highly disordered guest molecules in the pores was unsuccessful.

For that reason, in **1**, the contribution to the diffraction pattern from the disordered water molecules situated in the voids was subtracted from the observed data through the SQUEEZE method, implemented in PLATON.^36^ The total potential accessible voids calculated from PLATON is 9548.6 Å^3^ per unit cell which accounts for 55.0% of the unit cell volume [17355.0 Å^3^]. SQUEEZE estimated a total count of 4918 electrons per 8058.8 Å^3^ of solvent accessible volume in **1**, which is in good agreement to 123 water molecules (*Z* = 4; H_2_O = 10e^–^; 4918e^–^/4 = 1229.5e^–^ ≈ 123H_2_O). This estimated solvent amount must be added to the number of water molecules modeled and surrounding Ca^2+^ that is 37 per formula, to give a final formula of Ca_5_Cu_10_(aspartamox)_5_·160 H_2_O.

The hydrogen atoms of the ligand were set in calculated positions and refined as riding atoms, whereas, for water molecules, they were neither found nor calculated.

A summary of the crystallographic data and structure refinement for the crystal structure is given in Table S1. CCDC reference number is 2075709.

The final geometrical calculations on free voids and the graphical manipulations were carried out with PLATON^36^ implemented in WinGX,^41,42^ and CRYSTAL MAKER programs,^43^ respectively.

### Cell Cultures

MCF7 and SkBr3 breast cancer cells were maintained in DMEM/F-12 and RPMI 1640, respectively, supplemented with 5% fetal bovine serum (FBS), 100 mg/mL penicillin/streptomycin, and 2 mM l-glutamine (Life Technologies, Milan, Italy).

### HPLC-UV Analyses

The HPLC-UV analyses were performed by means of a FractionLynx system from Waters (Milford, MA) working in analytical mode. The instrument is equipped with a 2535 quaternary pump and a 2989 UV/visible detector. The analytical column used for the chromatographic separation was a C18 reversed-phase column, named Luna (250 × 4.6 mm, 5 μm, Phenomenex). The injection volume was 20 μL of a suitably diluted sample coming from solution in contact with MOF after 0, 1.5, 24, 48, and 120 h. The elution was carried out with 0.1% formic acid in water (solvent A) and methanol (solvent B) under gradient conditions. The gradient steps were the following: from 100 to 92% A (0–8 min), from 92 to 20% A (8–18 min), 20% A in isocratic for 2 min, from 20 to 80% A (20–24 min). Finally an isocratic flow (8 min) to equilibrate the system before starting the new analysis was used. The total run time was 32 min, while the flow rate was set at 1 mL/min and the UV detector was set at 280 nm. The concentration of the solution of dopamine hydrochloride in contact with MOF was evaluated using an external calibration curve gained by standard solutions ranging from 50 to 300 μg/mL. The experiment was performed in triplicate, and results are reported as average values ±3 SD. Data are reported in Figures 6 and S10.

### ICP-MS Analyses

The Ca^2+^ concentrations during degradation at pH = 2 were determined by utilizing an inductively coupled plasma-mass spectrometer (ICP-MS iCAP TQ Thermo Fisher Scientific, USA) equipped with a Peltier cooled high purity quartz baffled cyclonic spray chamber, a concentric borosilicate glass nebulizer, a wide 2.5 mm internal diameter quartz injector, a nickel sample, and two skimmer cones with 1.1 and 0.5 mm diameter orifices, respectively. The ICP torch was a demountable single piece quartz torch. The samples were collected by a Thermo Scientific Autosampler Housing with a peristaltic pump equipped with three-stop flared PVC pump tubing. A multielement standard solution was used to calibrate the instrument using different analytical concentrations. Ultrapure water (18.3 MΩ cm, Arioso, Human Corporation, Korea) was used for the aqueous solution preparation. Aliquots of 100 μL were taken for the determination of calcium concentrations at fixed time intervals. Experiment was performed in triplicate, and results are reported as average values ±3 SD. Data are reported in Figure S11.

## Supporting Information Available

Additional preparations and physical characterization data. Additional figures (Figures S1–S11). Crystallographic details and refinement for **1** (Table S1) (PDF)

## Accession Codes

CCDC 2075709 contains the supplementary crystallographic data for this paper. These data can be obtained free of charge via www.ccdc.cam.ac.uk/data_request/cif, or by emailing data_request@ccdc.cam.ac.uk, or by contacting The Cambridge Crystallographic Data Centre, 12 Union Road, Cambridge CB2 1EZ, UK; fax: +44 1223 336033.
