# Supplementary material for: A Biocompatible Aspartic-Decorated Metal–Organic Framework with Tubular Motif Degradable under Physiological Conditions
Source: Inorg Chem. 2021 Sep 2;60(18):14221–9. doi: 10.1021/acs.inorgchem.1c01701 (PMC8456407; doi:10.1021/acs.inorgchem.1c01701)
Supplement: Supplementary file 1 — ic1c01701_si_001.pdf [file ic1c01701_si_001.pdf]

Supporting Information (SI) for the manuscript:

A Biocompatible Aspartic-Decorated Metal-Organic Framework with Tubular Motif Degradable under Physiological Conditions

Marta Mon,[†] Rosaria Bruno,[§] Rosamaria Lappano,[#] Marcello Maggiolini,[#] Leonardo Di Donna,[§] Jesús Ferrando-Soria,[†,*] Donatella Armentano,[§,*] and Emilio Pardo[†,*]

[†]Departament de Química Inorgànica, Instituto de Ciencia Molecular (ICMOL), Universitat de València, 46980 Paterna, València, Spain

[§]Dipartimento di Chimica e Tecnologie Chimiche, Università della Calabria, Rende 87036, Cosenza, Italy

#Dipartimento di Farmacia e Scienze della Salute e della Nutrizione, Università della Calabria, Rende 87036, Cosenza, Italy

To whom correspondence should be addressed: emilio.pardo@uv.es; donatella.armentano@unical.it; jesus.ferrando@uv.es.

Table S1. Summary of Crystallographic Data for **1**.

Compound	**1**
Formula	$C_{50}Cu_{10}H_{350}N_{10}O_{210}Ca_5$
M (g mol^{-1})	5289.18
λ (Å)	0.71073
Crystal system	Orthorhombic
Space group	$P2_12_12_1$
a (Å)	23.196(2)
b (Å)	23.760(2)
c (Å)	31.489(3)
V (Å^3)	17355(3)
Z	4
ρ_{calc} (g cm^{-3})	2.024
μ (mm^{-1})	1.523
T (K)	90
θ range for data collection (°)	1.074 to 26.376
Completeness to θ = 25.0	100%
Measured reflections	356653
Unique reflections (Rint)	35318 (0.0895)
Observed reflections [$I > 2\sigma(I)$]	27409
Goof	1.001
Absolute structure parameter (Flack)	0.045(5)
R^a [$I > 2\sigma(I)$] (all data)	0.0640 (0.0786)
wR^b [$I > 2\sigma(I)$] (all data)	0.1713 (0.1799)
Largest diff. peak and hole	1.315 and -0.751 e.Å^{-3}
CCDC Deposition Number	2075709

^{a} $R = \sum(|F_o| - |F_c|)/\sum|F_o|$. ^{b} $wR = [\sum w(|F_o| - |F_c|)^2/\sum w|F_o|^2]^{1/2}$.

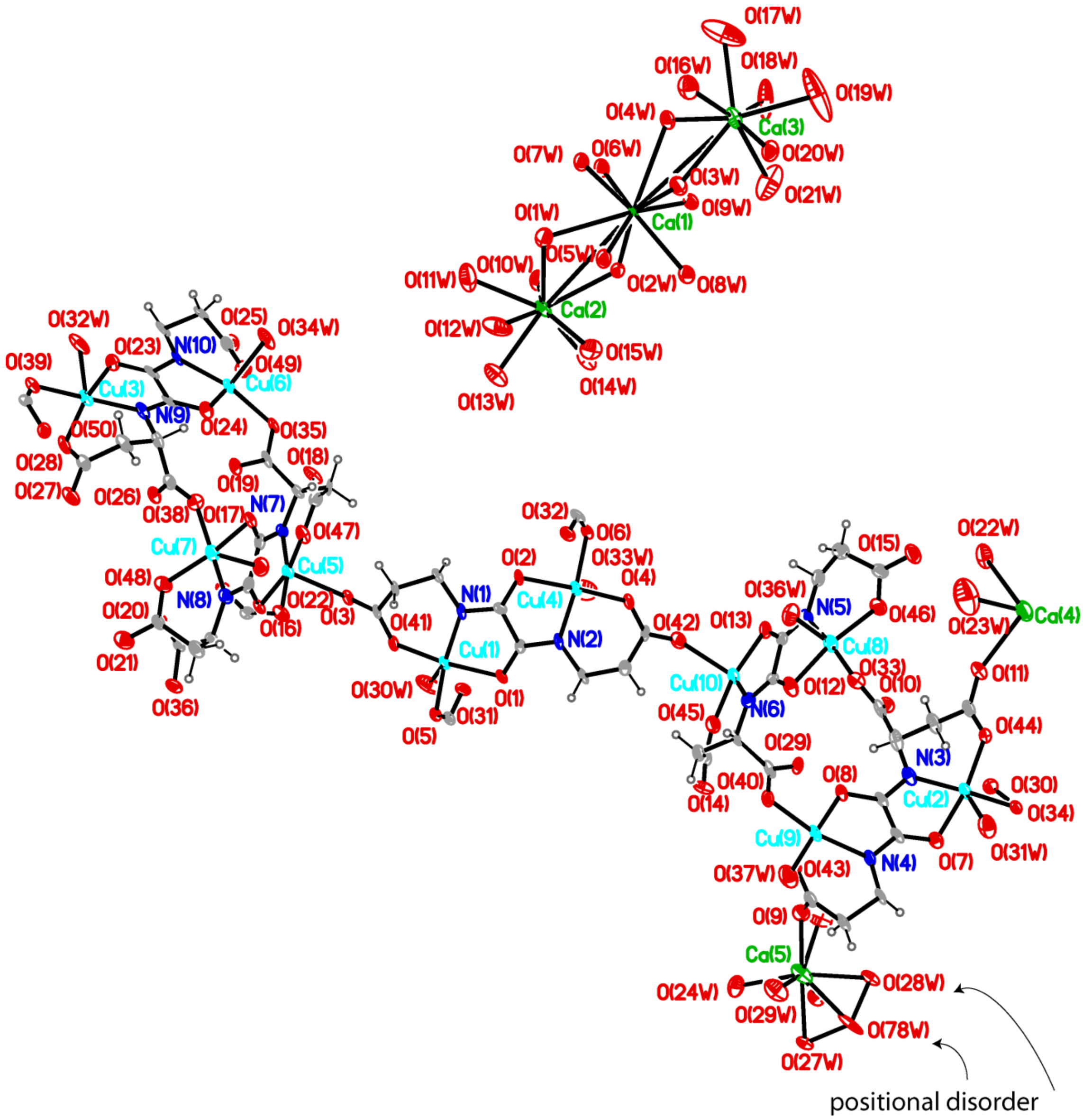

Figure S1. Perspective view of asymmetric unit in **1** showing atom numbering scheme. Thermal ellipsoid have been depicted at 50% of probability level.

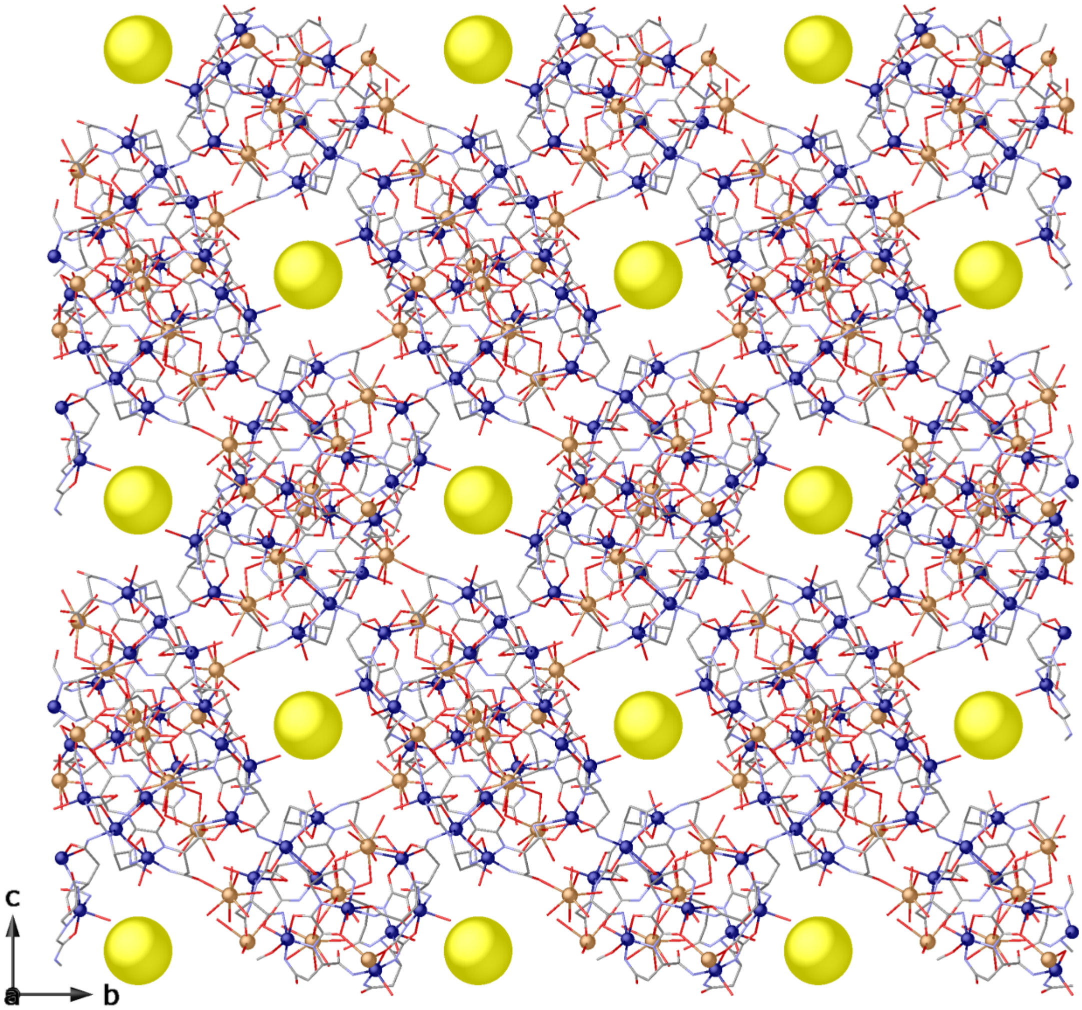

Figure S2. View along *a* crystallographic axis of hydrophilic channels exhibiting an almost squared shape with 0.4 nanometers as virtual diameter. Color code: copper, blue spheres; calcium, brown spheres; oxygen, red sticks; nitrogen, light blue sticks; carbon, grey sticks. Voids have been underlined by yellow spheres. Hydrogen atoms and lattice water molecules have been omitted for clarity.

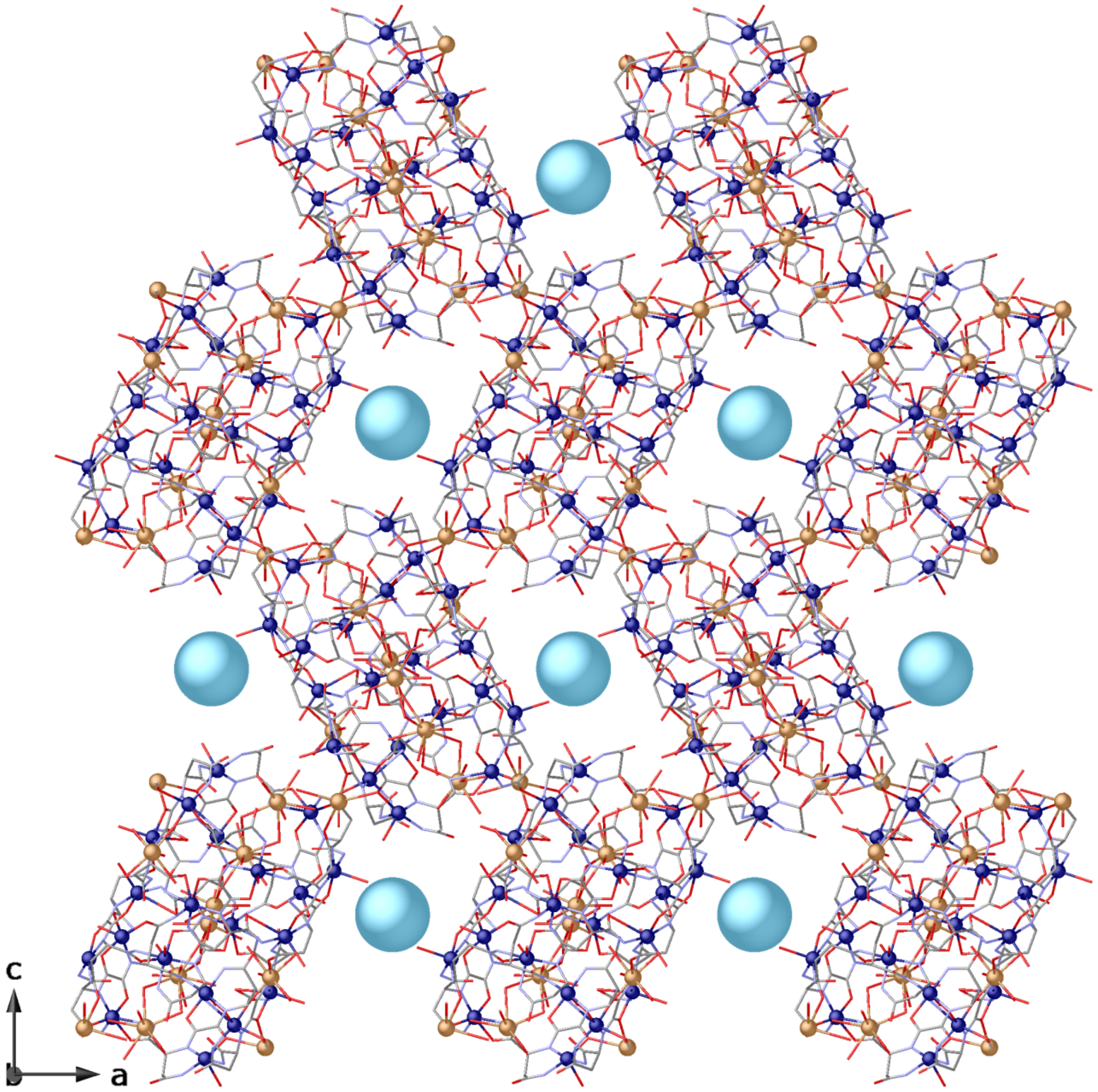

Figure S3. View along *b* crystallographic axis of hydrophilic channels exhibiting an irregular shape with 0.6 nanometers as virtual diameter. Color code: copper, blue spheres; calcium, brown spheres; oxygen, red sticks; nitrogen, light blue sticks; carbon, grey sticks. Voids have been underlined by light blue spheres. Hydrogen atoms and lattice water molecules have been omitted for clarity.

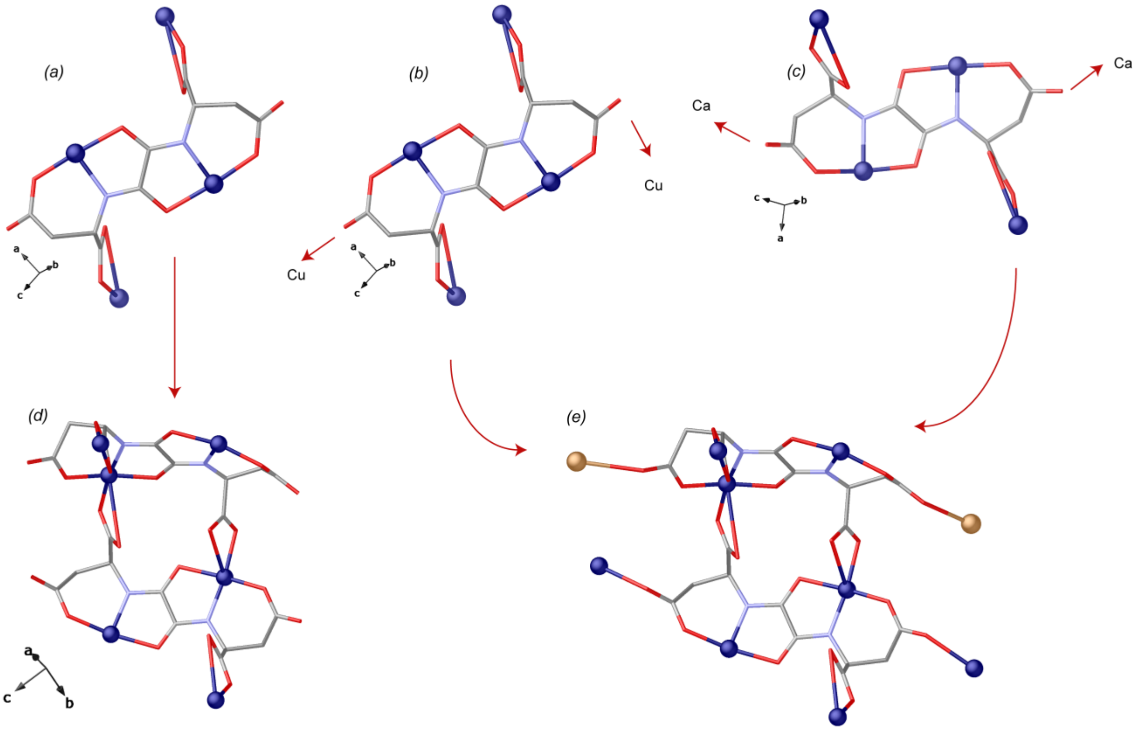

Figure S4. Details on coordination modes exhibited by aspartamox ligand in **1** where, notably, not all carboxyl 'free' groups act as linkers towards Cu^{2+} or Ca^{2+} ions with a portion of them available for supramolecular interactions. Color code as in Figure S2.

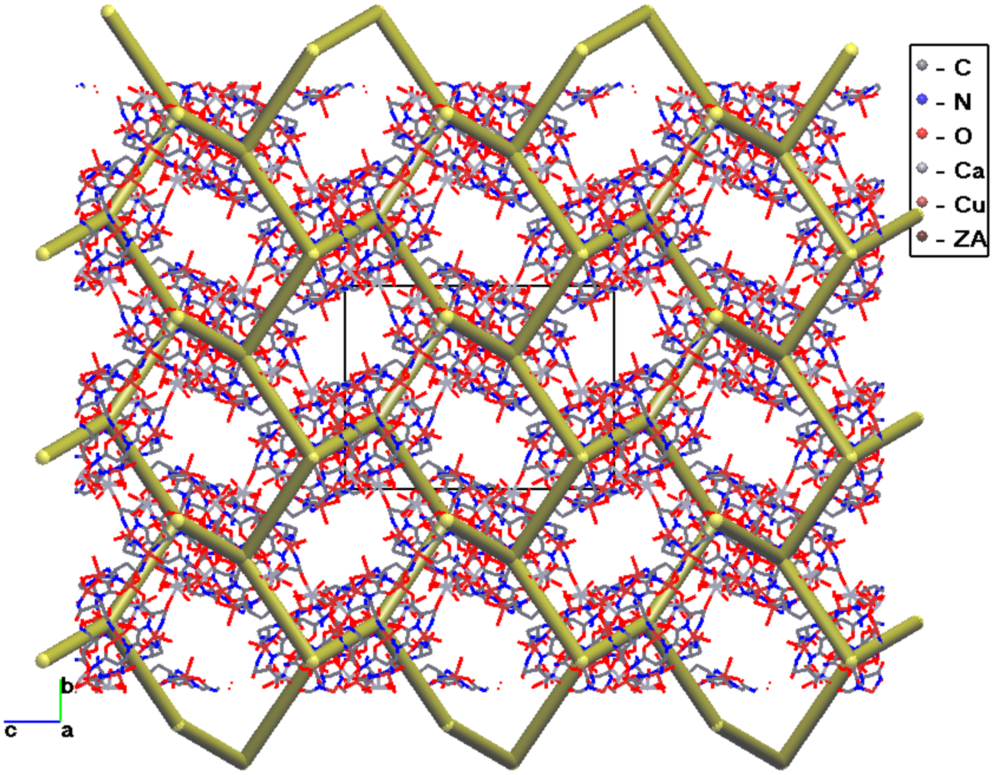

Figure S5. Schematic view along *a* axis, of the **dia** net topology in **1**, with details about deconstructed nodes.

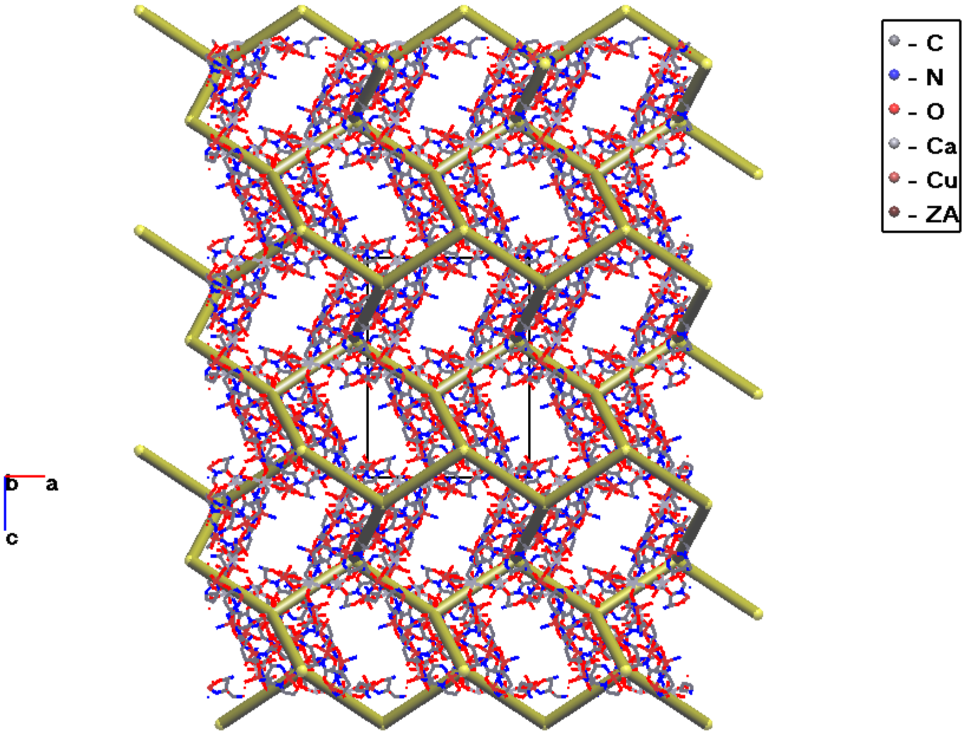

Figure S6. Schematic view along *b* axis, of the **dia** net topology in **1**, with details about deconstructed nodes.

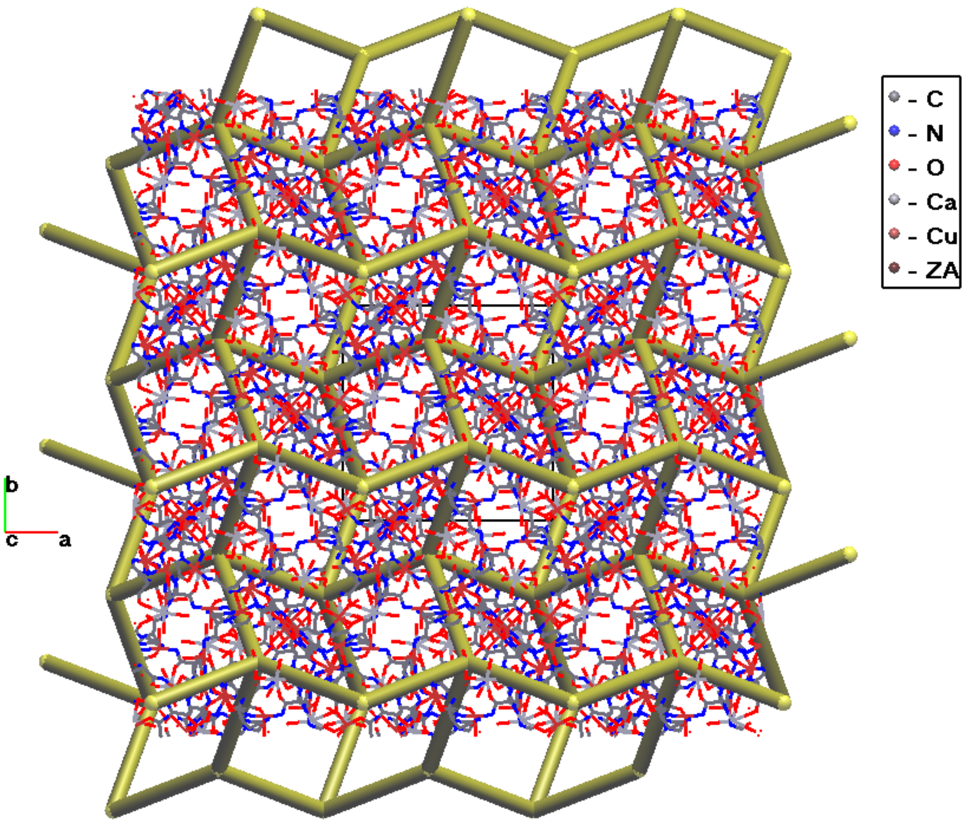

Figure S7. Schematic view along *c* axis, of the **dia** net topology in **1**, with details about deconstructed nodes.

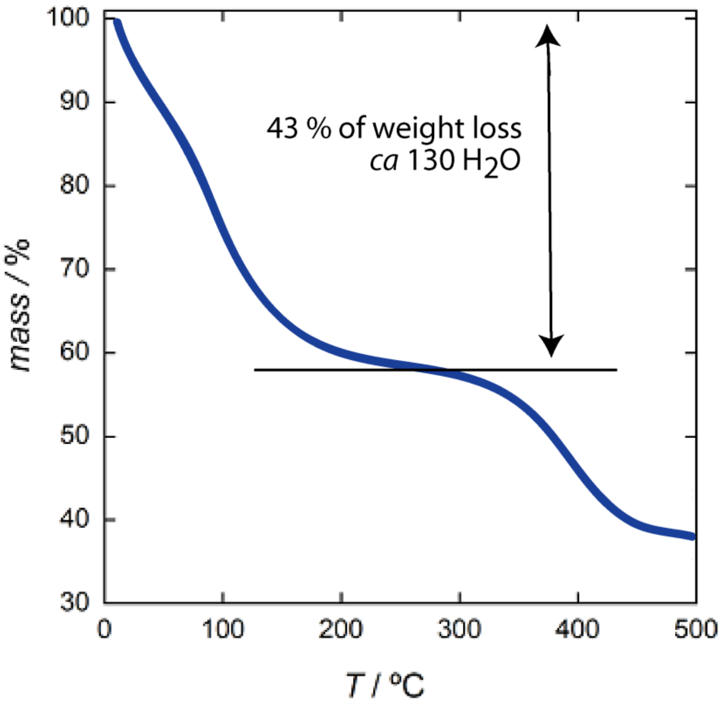

Figure S8. Thermo-Gravimetric analysis (TGA) of **1** under dry N_2 atmosphere.

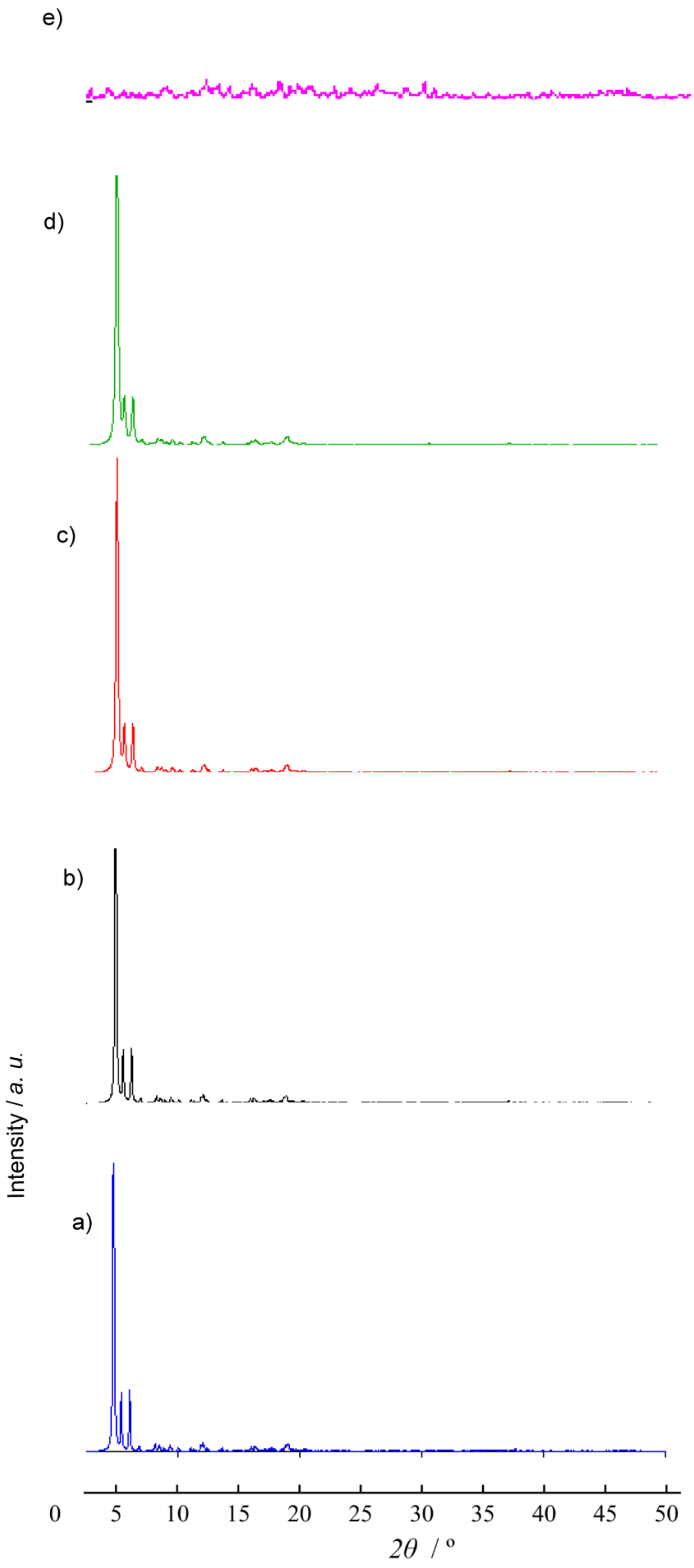

Figure S9. PXRD pattern profiles of **1** (in the 2θ range 2.0–50.0°). a) Calculated (blue) b) experimental (black) PXRD pattern profile on fresh sample of **1**. Variable pH and contact time with aqueous solutions XRD patterns at room temperature of **1**. After fifteen days immersed in a pure aqueous solution (red) (pH = 6.5 pure water at air) (c); after 48 h immersed in a pH = 11 aqueous solution (green) (d); after 48 h immersed in a pH = 3 aqueous solution (purple) (e).

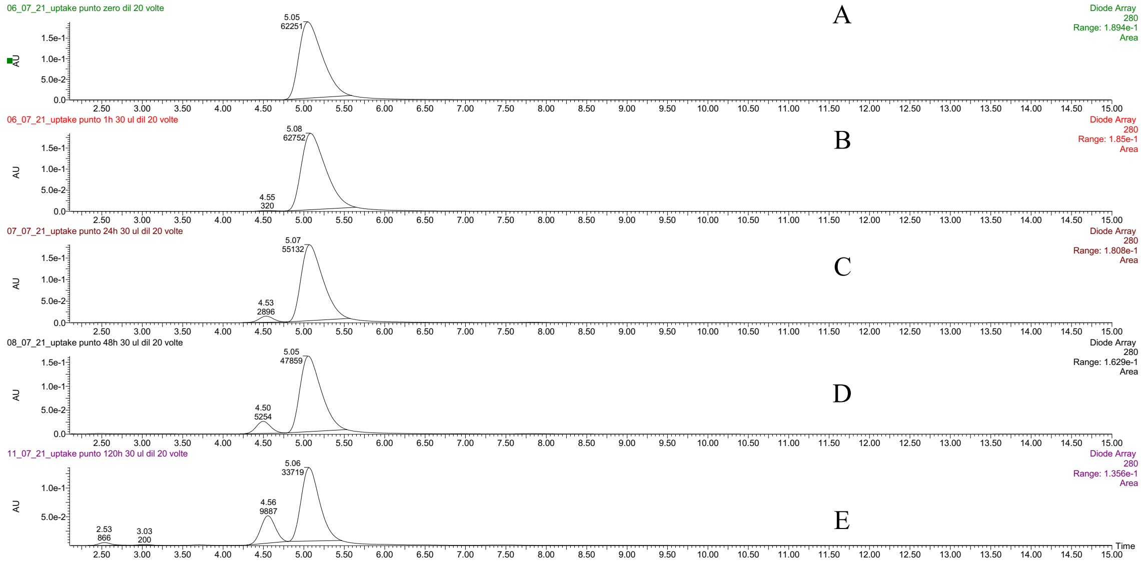

Figure S10. HPLC/UV chromatograms of the contact solution of dopamine hydrochloride at (A) t = 0 h, (B) t = 1.5 h, (C) t = 24 h, (D) t = 48 h and (E) t = 120 h, recorded at 280 nm.

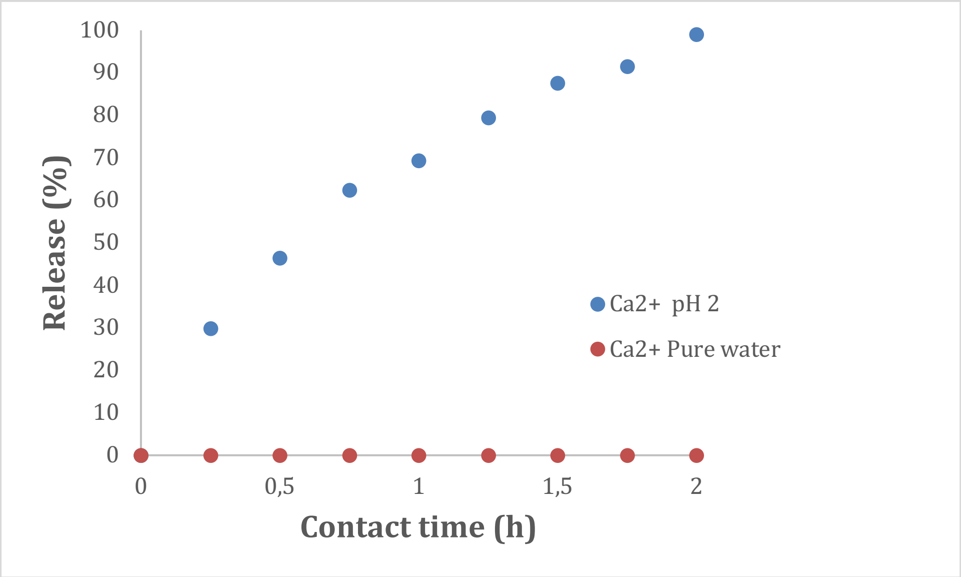

Figure S11. Percentage of delivery of Ca^{2+} in acidic aqueous media (pH = 2) and ultrapure water from **1** in the range 0-2 hours.
